# In silico development and experimental validation of a novel 7-gene signature based on PI3K pathway-related genes in bladder cancer

**DOI:** 10.1007/s10142-022-00884-2

**Published:** 2022-07-28

**Authors:** Linhui Wang, Yutao Wang, Jianbin Bi

**Affiliations:** Department of Urology, China Medical University, The First Hospital of China Medical University, Shenyang, Liaoning China

**Keywords:** Bladder urothelial cancer, PI3K pathway, Prognostic marker, CDK6

## Abstract

Although bladder cancer (BLCA) is the 10th most common tumor worldwide, particularly practical markers and prognostic models that might guide therapy are needed. We used a non-negative matrix factorization algorithm to classify PI3K pathway-related genes into molecular subtypes. A weighted gene co-expression network analysis (WGCNA) was generated to identify co-expression modules. Univariate Cox regression, least absolute shrinkage sum selection operator-Cox regression, and multivariate Cox regression were utilized to develop a prognostic score model. Kaplan–Meier analysis and receiver operating characteristics were utilized to measure the model’s effectiveness. A nomogram was constructed to improve the predictive ability of the model based on clinical parameters and risk. Decision curve analysis (DCA) was used to evaluate the nomogram. To evaluate the immune microenvironment, an estimate algorithm was used. Drug sensitivity was identified using the R package “pRRophetic.” UM-UC-3 cell line was used to measure the effect of CDK6 in Western blotting, proliferation assay, and 5-ethynyl-20-deoxyuridine assay. Based on PI3K pathway-related genes, The Cancer Genome Atlas (TCGA)-BLCA and GSE32894 patients were divided into two subtypes. Twenty-five co-expression modules were established using the WGCNA algorithm. A seven-gene signature (CDK6, EGFR, IGF1, ITGB7, PDGFRA, RPS6, and VWF) demonstrated robustness in TCGA and GSE32894 datasets. Expression levels of CDK6 and risk positively correlated with M2 macrophages and IgG. Cisplatin, gemcitabine, methotrexate, mitomycin C, paclitaxel, and vinblastine are sensitive to different groups based on the expression of CDK6 and risk. Functional experiments suggested that CDK6 promotes the proliferation of UM-UC-3 cells. We constructed a seven-gene prognostic signature as an effective marker to predict the outcomes of BLCA patients and guide individual treatment.

**Supplementary Information:**

The online version contains supplementary material available at 10.1007/s10142-022-00884-2.

## Introduction

The world’s 10th leading cause of tumor-related mortality and morbidity is bladder cancer (BLCA) (Shinde-Jadhav et al. 2021), divided into muscle and non-muscle-invasive BLCA. Despite treatment advances, the diagnostic yield, outcomes, and 5-year survival rate have hardly changed (Berdik 2017). BLCA carries the highest recurrence rate, 50–70% of patients with incomplete cystectomy relapse within 5 years of treatment (Ainsworth 2017). Therefore, it is critical to identify markers to improve the diagnostic yield and outcome.

The PI3K signaling pathway is associated with the proliferative capacity of tumor cells (Pollak 2018). PI3K pathway-related genes were found to be significantly altered in many cancers, and these altered genes can promote cancer growth, apoptosis, and metastasis in pancreatic cancer (Banh et al. 2020), breast cancer (Garcia-Martinez et al. 2021), melanoma (Hamm et al. 2021), and BLCA (Hsieh et al. 2011). Studies focused on how the PI3K pathway affects the proliferation ability of BLCA cells as a downstream pathway regulated or modified by upstream molecules. However, there is no study on the effects of the PI3K pathway on treatment and outcome. The rapid development of gene sequencing and the widespread availability of public databases have enabled the collection of gene expression data and the identification of biomarkers.

In the current study, TCGA (https://cancergenome.nih.gov/) and the Gene Expression Omnibus (GEO, http://www.ncbi.nlm.nih.gov/geo/) databases were used to obtain gene expression data and clinical information from BLCA patients. Using a univariate Cox proportional hazard regression model, genes that predict outcome were selected, and a prognostic model for BLCA was established using a least absolute shrinkage and selection operator (LASSO) regression. This model was subsequently validated in four cohorts.

## Methods

### PI3K pathway-related genes

The list of genes associated with the human PI3K pathway was downloaded from the Kyoto Encyclopedia of Genes and Genomes (KEGG) (Kanehisa and Goto 2000), and 275 genes were included.

### Obtaining and processing TCGA and GEO data

The BLCA cohort downloaded from TCGA included 430 samples: 411 tumor samples and 19 normal tissues. The gene expression matrix (TPM) and clinical information for each sample were applied for subsequent analysis. All samples in the TCGA-BLCA cohort served as a training set to construct the prognostic model. RNA expression profiles were normalized by log2(exp + 1). The GSE32894 (Sjödahl et al. 2012) cohort with 224 samples was obtained from the GPL6947 platform from GEO. Data from TCGA and GEO cohorts were normalized together by the “Combat” function, using the R package “sva.” All clinical information for patients in the TCGA and GSE32894 cohorts was presented in Table 1.

**Table 1:** Basic clinical information for the two cohorts

Clinical factors	TCGA_BLCA		GSE32894	
	*n* = 430	%	*n* = 224	%
Stage				
I-II	137	31.86	—	—
III-IV	291	67.67	—	—
T				
Ta–T2	—	—	216	96.43
T3–T4	—	—	8	3.57
Age				
≤ 60	113	26.28	46	20.54
> 60	317	73.72	178	79.46
Gender				
Male	314	73.02	163	72.77
Female	116	26.98	61	27.23
Grade				
Low	21	4.88	—	
High	406	94.42	—	
Vital status				
Alive	237	55.12	199	88.84
Dead	193	44.88	25	11.16
Follow-up (mean ± SD)				
(year)	2.16 ± 2.24		3.28 ± 2.10	

### Molecular typing based on PI3K pathway-related genes

Fifty-eight genes were not found when extracting the expression of 275 PI3K pathway-related genes from the TCGA cohort and GSE32894 cohort; therefore, the expression of 217 genes was used for subsequent studies. To conduct a univariate Cox analysis, the *coxph* function was utilized. The “brunet” criterion and the non-negative matrix factorization (NMF) algorithm with 50 iterations were used to cluster BLCA samples. The clusters’ *k* number was between two and 10. To determine the average profile width of the common membership for each subclass with a minimum membership of 10, the R package NMF was utilized. The clusters’ optimal numbers were determined using comprehensive consideration of dispersion, cophenetic, and silhouette.

### WGCNA

Using the R package “WGCNA,” a co-expression algorithm was employed to identify co-expressed genes and divide the genes into multiple co-expression modules based on the protein-coding genes’ expression in the TCGA-BLCA (Langfelder and Horvath 2008). We constructed a scale-free co-expression network with the soft threshold = 5 and *R*^2^ = 0.87. The minimum module’s number of genes was set to 30.

### Functional analysis

To identify pathways and biological functions enriched by co-expression module genes, KEGG (https://www.genome.jp/kegg/) (Kanehisa and Sato 2020) and Gene Ontology (GO) (http://geneontology.org/) (Ashburner et al. 2000) analysis was utilized. The R packages “KEGG” and “GO” were used.

### Establishment and verification of the prognostic score model

The R package “survival” was used to perform a univariate Cox regression to identify genes linked with outcome in TCGA-BLCA. The genes’ numbers in the model were then reduced using the LASSO-Cox algorithm (Tibshirani 1997) using the R package “glmnet.” Multivariate Cox regression was used to establish a prognostic score model, using the R package “survival.” We divided TCGA-BLCA patients into high- or low-risk subgroup based on the median of risk values. Based on the median of the risk values obtained from the TCGA-BLCA cohort, we divided GSE32894 patients into high- or low-risk subgroup. The predictive ability of our model was validated using Sangerbox (http://sangerbox.com/), a tool for bioinformatic data analysis based on the R language.

### Establishment of nomogram and DCA

The R package “rms” and “regplot” (Zhang and Kattan 2017) was used to establish a nomogram combined with clinical data and risk score. The nomogram can predict BLCA patients’ 1-, 3-, and 5-year survival rates and improve the predictive ability of the constructed model. The predictive ability of the nomogram was evaluated by calibration and DCA, using the R package “ggDCA.”

### Immune microenvironment analysis

The ESTIMATE algorithm (Yoshihara et al. 2013) is used to transform gene expression data from each patient into the fractions of stromal and immune cells, thereby obtaining stromal and immune scores. To determine the correlation of risk score and gene expression with tumor purity, stromal score, immune score, and various inflammatory factors, the R package “heatmap” was used. Correlations between immune cells and risk score or gene expression were calculated using the R package “pheatmap.”

### Drug sensitivity analysis

Gene expression data were transformed into drug sensitivity data. Then, using the R package “pRRophetic,” relationships were determined between risk score and gene expression or drug sensitivity. The R package “ggpubr” was used to draw boxplots to display results.

### Cell culture and transfection

The Chinese Academy of Sciences Cell Bank (China) provided the UM-UC-3 human bladder cell line. UM-UC-3 cells were cultured in high-glucose DMEM (Hyclone) with 10% fetal bovine serum (Gibco), at 37 ℃ and 5% CO_2_. The small interfering RNAs (siRNA) that reduce CDK6 expression were acquired from JTSBIO Co. (China). The sequences of Si1-CDK6 were as follows: sense: AGUUAGUUUGGUUUCUCUGUC; anti-sense: CAGAGAAACCAAACUAACUUU. The sequences of Si2-CDK6 were as follows: sense: AACACUAAAGUUAGUUUGGUU; anti-sense: CCAAACUAACUUUAGUGUUUG.

### Western blotting

Radioimmunoprecipitation assay (RIPA) buffer was used to take protein from cells. A bicinchoninic acid assay kit measured the concentrations of protein. Different protein bands were separated by 10% sodium dodecyl sulfate–polyacrylamide gel electrophoresis, then transferred to polyvinylidene fluoride membranes. After blocking the membranes, they were incubated overnight with primary antibodies. The membranes were washed and then incubated with secondary antibodies for 1 h. Finally, membranes were rewashed, and the enhanced chemiluminescence detected the protein expression. ImageJ analyzed the results of pictures.

### Cell proliferation assay

UM-UC-3 cells were plated in 96-well plates. Cell Counting Kit‐8 assay reagent (Bimake, USA) was added to each pore to be measured according to the manufacturer’s instructions. Absorbance was measured on an automated reader (Bio-Rad) at 450 nm.

### EdU assay

UM-UC-3 cells were plated in 24-well plates. EdU assay reagent (Beyotime Biotechnology, China) was added to each pre according to the manufacturer’s instructions. A fluorescent microscope (Olympus Corporation, Japan) was used to obtain images, and the number of proliferating cells was counted using ImageJ software.

### Statistical analysis

All significance tests of differences were performed using R software (Rx64 4.1.2). All R packages were obtained From BioConductor (http://www.bioconductor.org) or CRAN (https://cran.r-project.org). *P* < 0.05 was considered significant.

## Results

### Molecular subtype identification using the NMF algorithm

**Fig. 1:**
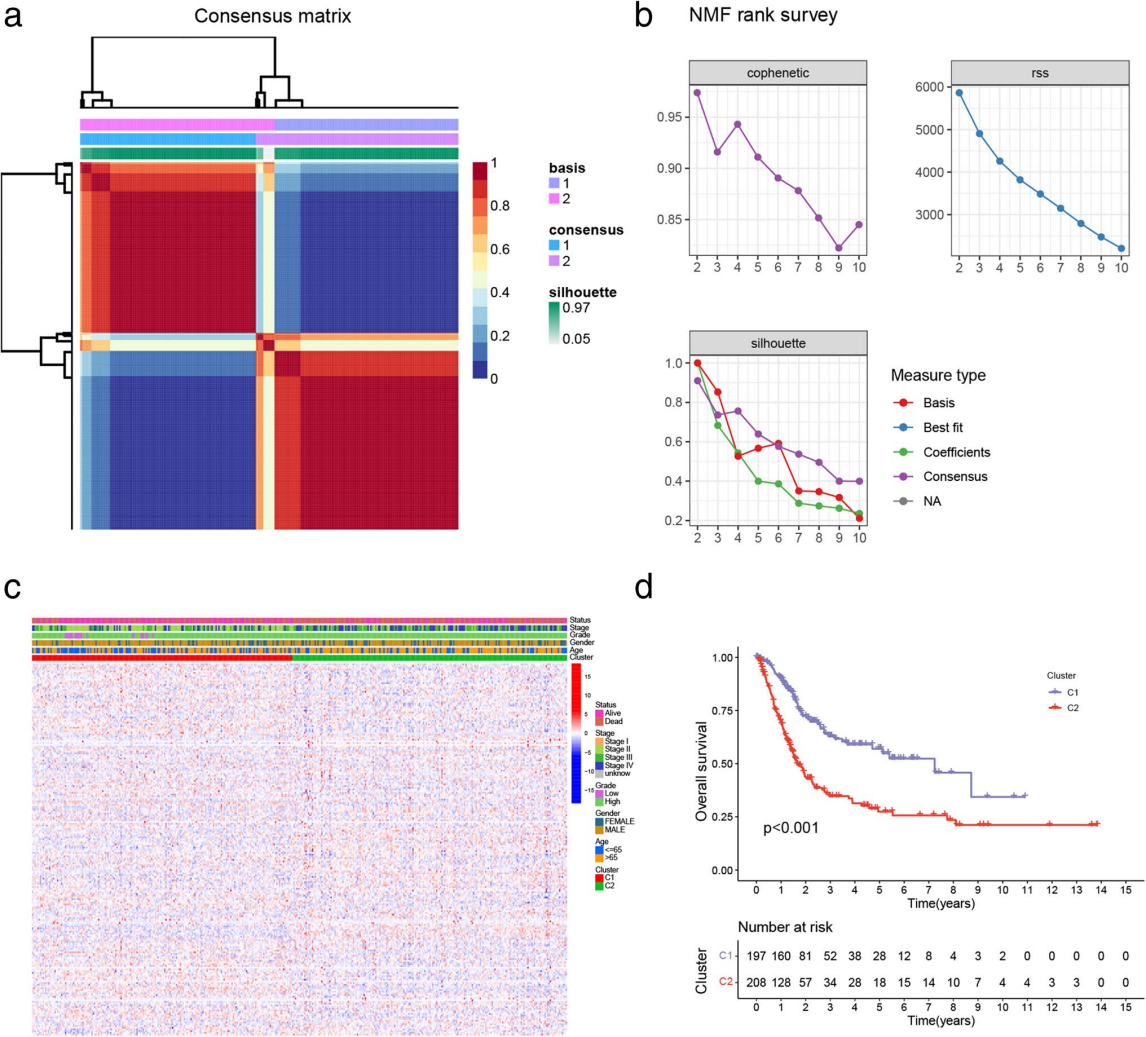
Non-negative matrix factorization (NMF) analysis. **a** NMF clustering (*K* = 2) consensus map. **b** The dispersion, RSS, and cophenetic distributions when rank = 2–10. **c** Cluster heat map of 217 PI3K pathway-related genes. **d** The two subtypes overall survival

The “survival” R package was used to conduct a single-factor Cox analysis. The 55 genes with *P* < 0.01 associated with BLCA outcome were obtained. The “Brunet” criterion and the NMF algorithm with 50 iterations were used to cluster BLCA samples. The clusters’ *k* numbers were between two and 10. The average profile width of the common membership matrix was determined using the R package NMF, with a minimum membership of 10 for each subclass. The cluster groups’ optimal number (*k* = 2) was determined using cophenetic dispersion and silhouette (Fig. 1a, b). The expression levels of PI3K pathway-related genes for samples in each group are shown in Fig. 1c. The outcome was worse in the C2 group than in the C1 group (Fig. 1d). The NMF algorithm was validated in the GSE32894 cohort. In the GSE32894 cohort, the cluster groups’ optimal number was 2 (Fig. S1a), and the C2 group had a worse outcome than the C1 group (Fig. S1b).

### WGCNA and functional analysis of co-expression modules

**Fig. 2:**
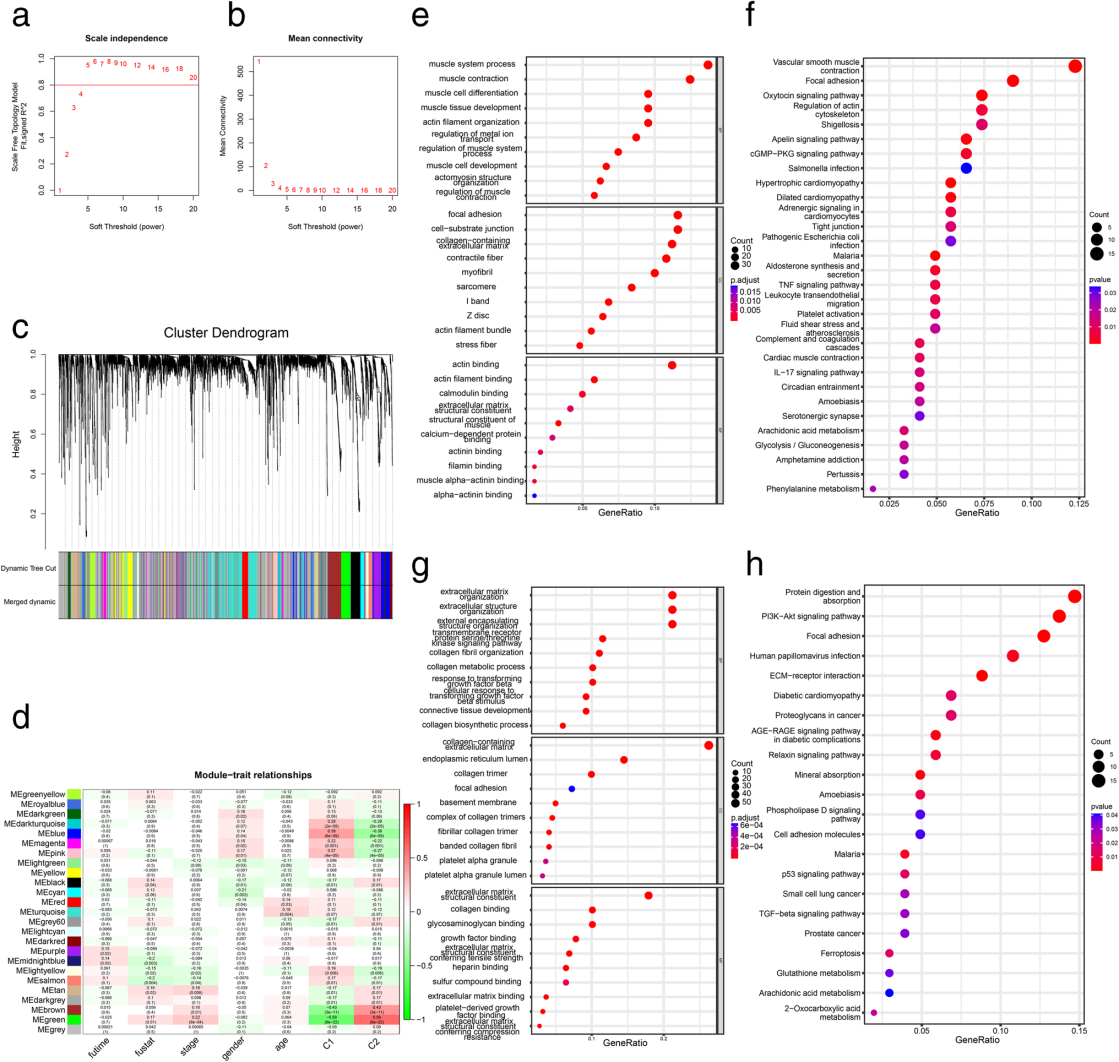
Weighted co-expression network and enrichment analysis. **a** Samples’ cluster analysis. **b** Network topology for various soft-thresholding power analysis. **c** Genes were divided into modules using the dynamic hybrid cutting method, and 25 co-expression modules were identified. **d** The correlation coefficients between modules and different phenotypes. **e** The brown module’s top 10 GO enrichment analyses (BP, CC, BF). **f** The brown module’s top 10 KEGG enrichment analyses. **g** The green module’s top 10 GO enrichment analyses (BP, CC, BF). **h** The green module’s top 10 KEGG enrichment analyses

The samples were clustered using hierarchical clustering based on the protein-coding genes’ expressions in TCGA-BLCA (Fig. 2a), and a topological overlap matrix was established (*β* = 5) (Fig. 2b). The co-expressed genes were grouped into a module using dynamic tree shearing; 25 co-expression modules were established (Fig. 2c). The correlation between each module and futime, fustat, stage, gender, age, cluster 1 and cluster 2 is shown in Fig. 2d. The highest correlations were between the brown and green modules and clusters. R software was used to perform KEGG pathway enrichment analysis and GO analysis on the genes of brown and green modules. Cellular component (CC), biological process (BP), and molecular function (BF) were included in GO terms. The top 10 GO terms of the brown module in each part of the GO analysis and the top 30 in the KEGG pathway enrichment analysis are displayed in Fig. 2e, f. The results of the green module are presented in Fig. 2g, h.

### Establishment of a prognostic risk model

**Fig. 3:**
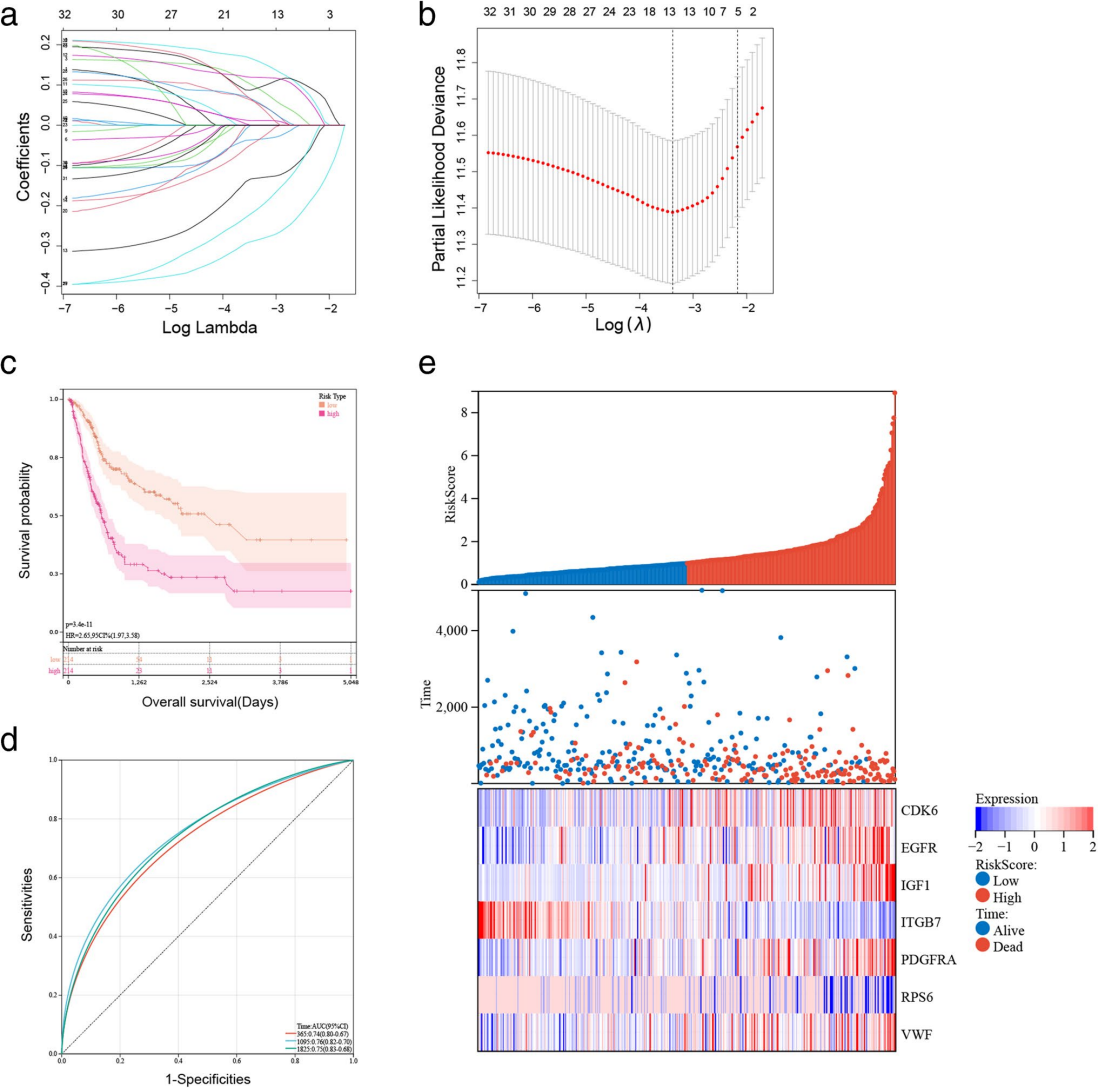
Establishment and effect of seven-gene signature in TCGA. **a** The coefficients of the LASSO-Cox regression analysis shrinkage. **b** Ten-fold cross-validation of the LASSO-Cox regression analysis. **c** Kaplan–Meier survival curves of high- and low-risk groups in TCGA (*P* < 0.001). **d** Receiver operating characteristic curves of the seven-gene model for predicting 1-, 3-, and 5-year survival in TCGA. **e** The distribution of the risk score, survival status, and expression of seven genes for each sample in TCGA

Genes associated with the TCGA-BLCA outcomes among PI3K pathway-related genes were selected using univariate Cox regression, and we identified 55 genes with *P* < 0.01. The LASSO-Cox algorithm was performed on 55 genes to reduce the number of the constructed risk score prognostic model (Fig. 3a, b); 11 genes (CDK6, CRTC2, EGFR, IGF1, IKBKB, IL7, ITGB7, LAMA2, NGF, PDGFRA, PPP2R3A, RPS6, and VWF) was screened. Finally, a risk model was constructed using multivariate Cox regression from seven genes. The signature formula for the seven mRNAs was as follows: RiskScore = 0.210687 307280298 × expCDK6 + 0.25722712750568  × expEGFR + 0.162230299646635 × e xpIGF1 – 0.436384884423358 × expITGB7 + 0.145930821644015 × expP DGFRA-0.294385373540532 × expRPS6  + 0.193651081566216 × expVWF.

The patients were divided into two risk groups. Survival curves were drawn to compare survival. Outcomes were worse in the high-risk group than in the low-risk group (*P* < 0.001) in Fig. 3c. Receiver operating characteristic (ROC) curves were drawn to determine the model’s applicability. Its 1-, 3-, and 5-year areas under the curve (AUCs) were 0.74, 0.76, and 0.75, respectively (Fig. 3d). Survival status, risk score, and expression of the seven genes for each sample are shown in Fig. 3e.

**Fig. 4:**
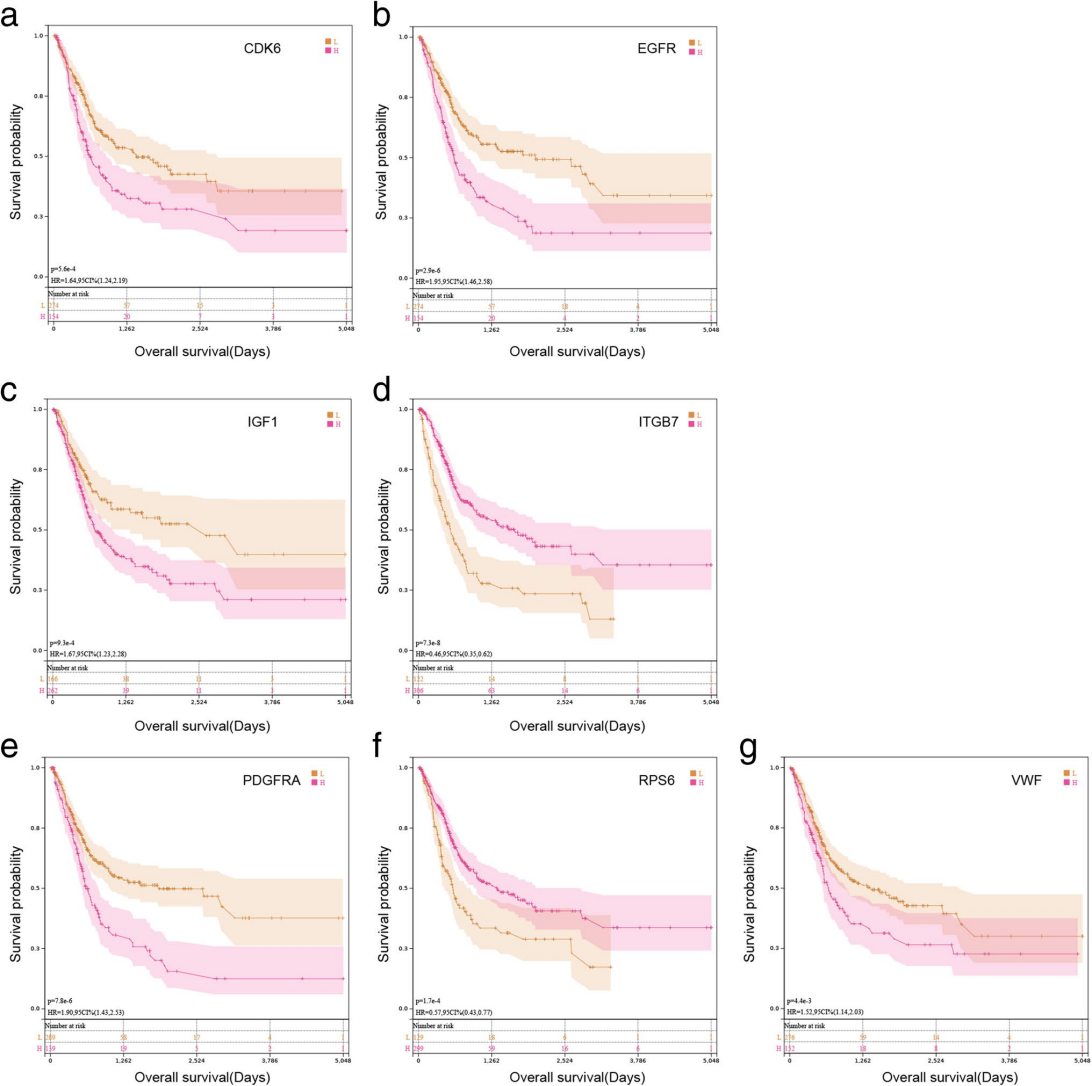
**a**–**g** Kaplan–Meier survival curves of high and low-expression groups based on the expression of seven genes in the model in TCGA

The seven factors were separately analyzed for Kaplan–Meier (KM) analysis to determine whether they could be independent factors to determine the BLCA outcome. CDK6 (*P* < 0.05), EGFR (*P* < 0.05), IGF1 (*P* < 0.05), ITGB7 (*P* < 0.05), PDGFRA (*P* < 0.05), RPS6 (*P* < 0.05), and VWF (*P* < 0.05) were identified (Fig. 4a–g); in GSE32894, CDK6 (*P* < 0.05), EGFR (*P* > 0.05), IGF1 (*P* > 0.05), ITGB7 (*P* < 0.05), PDGFRA (*P* < 0.05), RPS6 (*P* < 0.05), and VWF (*P* < 0.05) were identified (Fig. S2a-g).

### Robust verification of the risk signature

**Fig. 5:**
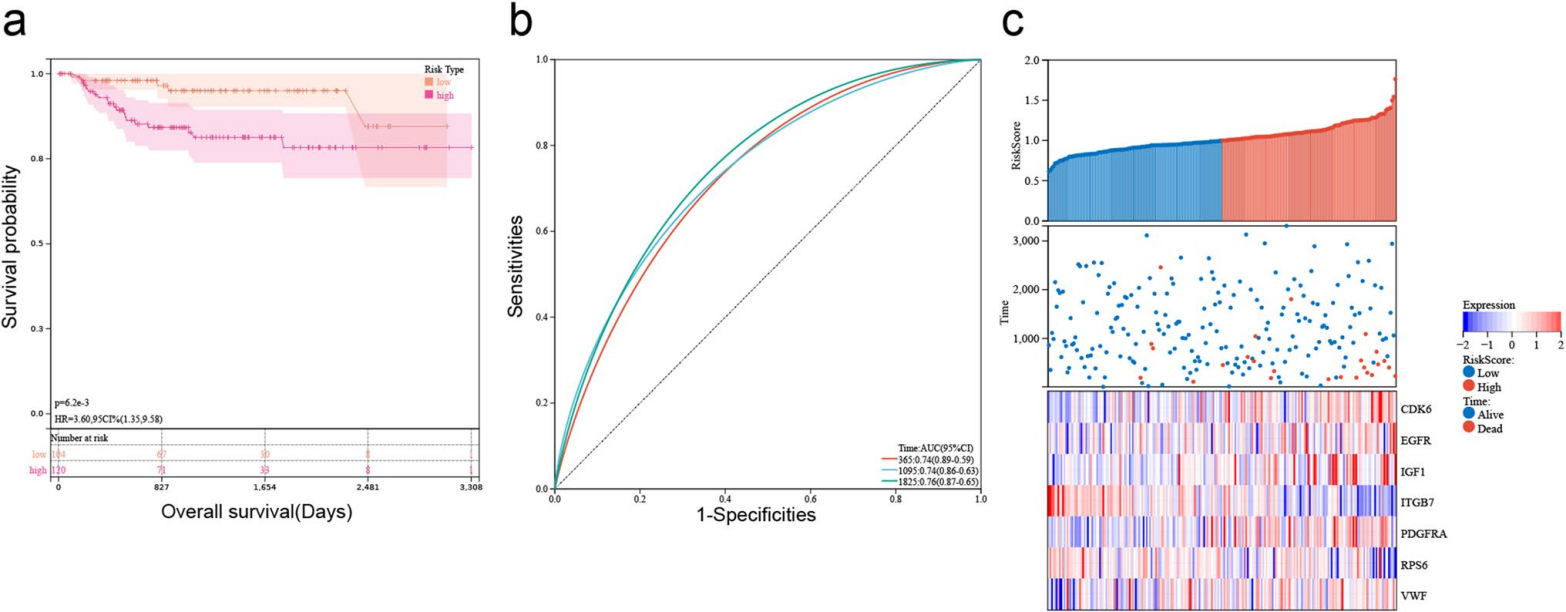
Validation of the seven-gene signature in GSE32894. **a** Kaplan–Meier survival curves of high- and low-risk groups (*P* < 0.001). **b** The seven-gene model’s ROC curves for predicting 1-, 3-, and 5-year survival. **c** The risk score distribution, survival status, and expression of seven genes for each sample

To assess the model’s applicability, risk scores of GSE32894 were calculated based on the risk model. The patients were divided into high- and low-risk groups. The survival curve of the external cohort was drawn to determine the model’s efficiency. Outcomes were worse in the high-risk group than in the low-risk group (*P* < 0.05) (Fig. 5a). In the GSE32894, the 1-, 3-, and 5-year AUCs were 0.74, 0.74, and 0.76, respectively (Fig. 5b). The risk, survival status distribution, and patients’ gene expression in the external cohort are shown in Fig. 5c. Univariable and multivariable Cox regression analysis showed that age, stage and risk score could independently predict the prognoses of BLCA patients (Table 2).

**Table 2:** Univariable and multivariable Cox regression analysis of clinical characteristics and risk score with overall survival in TCGA-BLCA

Variables	Univariable analysis				Multivariable analysis			
	HR	95% CI of HR		*P*	HR	95% CI of HR		*P*
		Lower	Upper			Lower	Upper	
Age	1.03	1.02	1.05	4.74E − 05	1.02	1.01	1.04	0.0062
Gender	0.86	0.62	1.19	0.3489	0.90	0.65	1.25	0.5327
Grade	2.89	0.72	11.69	0.1361	1.16	0.28	4.82	0.8369
Stage	1.74	1.44	2.11	1.71E − 08	1.57	1.28	1.92	1.05E − 05
RiskScore	1.61	1.46	1.77	5.51E − 22	1.50	1.35	1.66	1.24E − 14

### Analysis between risk models and clinical characteristics

**Fig. 6:**
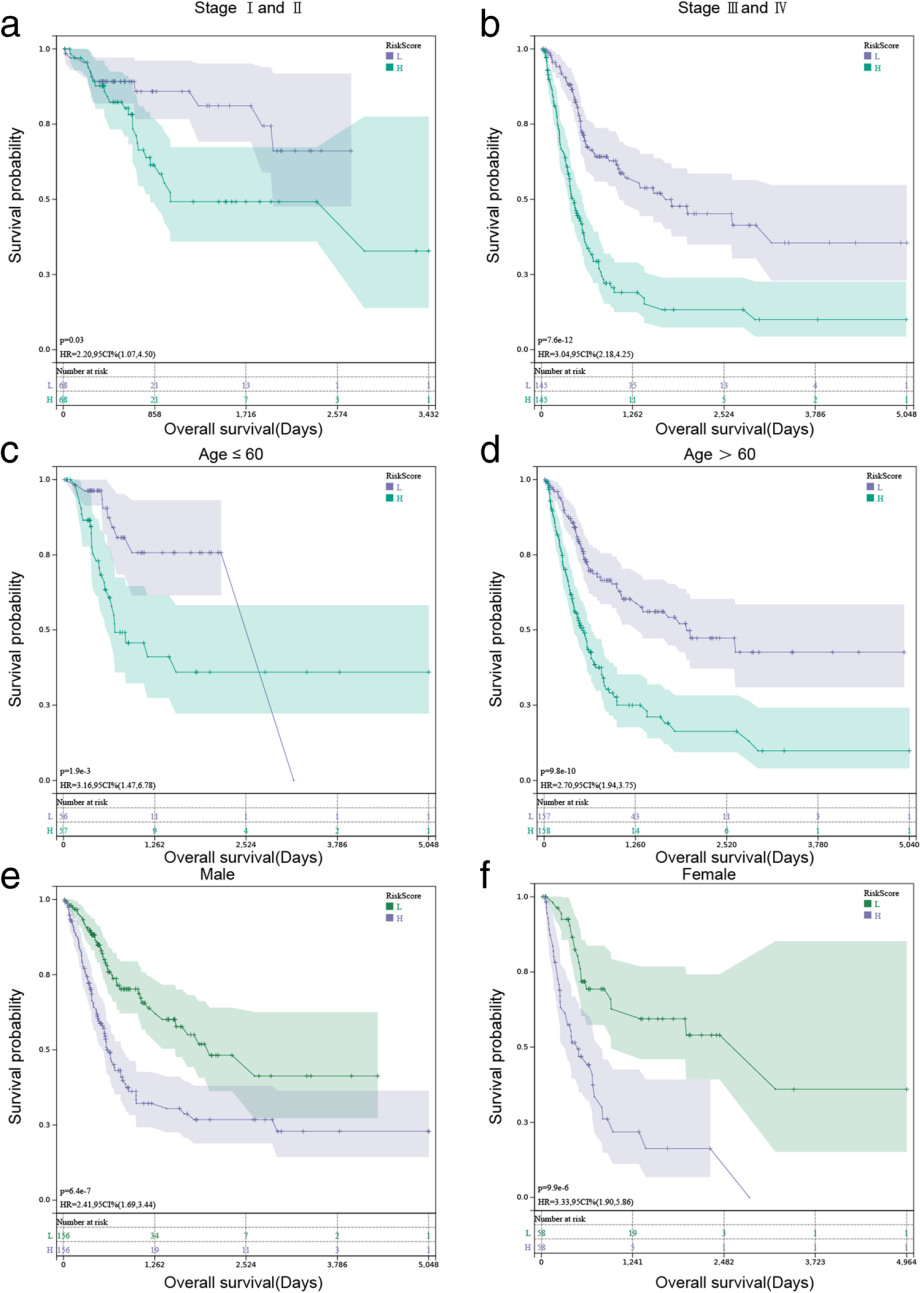
Kaplan–Meier (KM) survival analysis in different subgroups based on stage, age, and gender in TCGA. **a** KM survival curves of patients in stage I + II (*P* < 0.05). **b** KM survival curves of patients in stage III + IV (*P* < 0.001). **c** KM survival curves of patients aged ≤ 60 (*P* < 0.001). **d** KM survival curves of patients with aged > 60 (*P* < 0.001). **e** KM survival curves of male patients (*P* < 0.001). **f** KM survival curves of female patients (*P* < 0.001)

Patients were grouped according to their stage, age, and gender. To determine the model applicability, KM curves were drawn. TCGA findings demonstrated that the model predicted outcomes with stage I + II, stage III + IV, age ≤ 60, age > 60, male and female in Fig. 6a–f (*P* < 0.05 for all). The analysis of the GSE32894 cohort demonstrated that the model could predict outcomes with Ta-T2 (*P* < 0.05) and female (*P* < 0.05) (Fig. S3a–f).

### Construction and predictive ability of nomogram

**Fig. 7:**
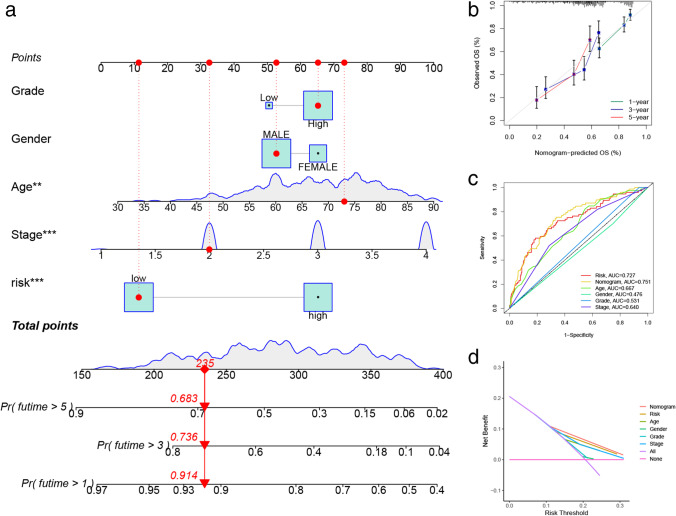
Construction and evaluation of nomogram. **a** Nomogram. **b** The nomogram calibration curves of 1-, 3-, and 5-year survival probabilities. **c** ROC curves. **d** DCA

Combining clinical parameters (grade, gender, age, and stage) and risk score, a prognostic nomogram was constructed with BLCA patients’ survival probability (Fig. 7a). The 1-, 3-, and 5-year calibration were used to evaluate the predictive discrimination of the nomogram, and the result showed that the nomogram had the best predictive discrimination for 1-year overall survival (Fig. 7b). The AUC value (0.751) of the nomogram was better than the constructed model (0.727), age (0.667), gender (0.476), grade (0.531), and stage (0.640), as shown in Fig. 7c. The DCA result showed that the net benefit of the nomogram was greater than the single independent clinical feature (Fig. 7d).

### Immune environment evaluation

**Fig. 8:**
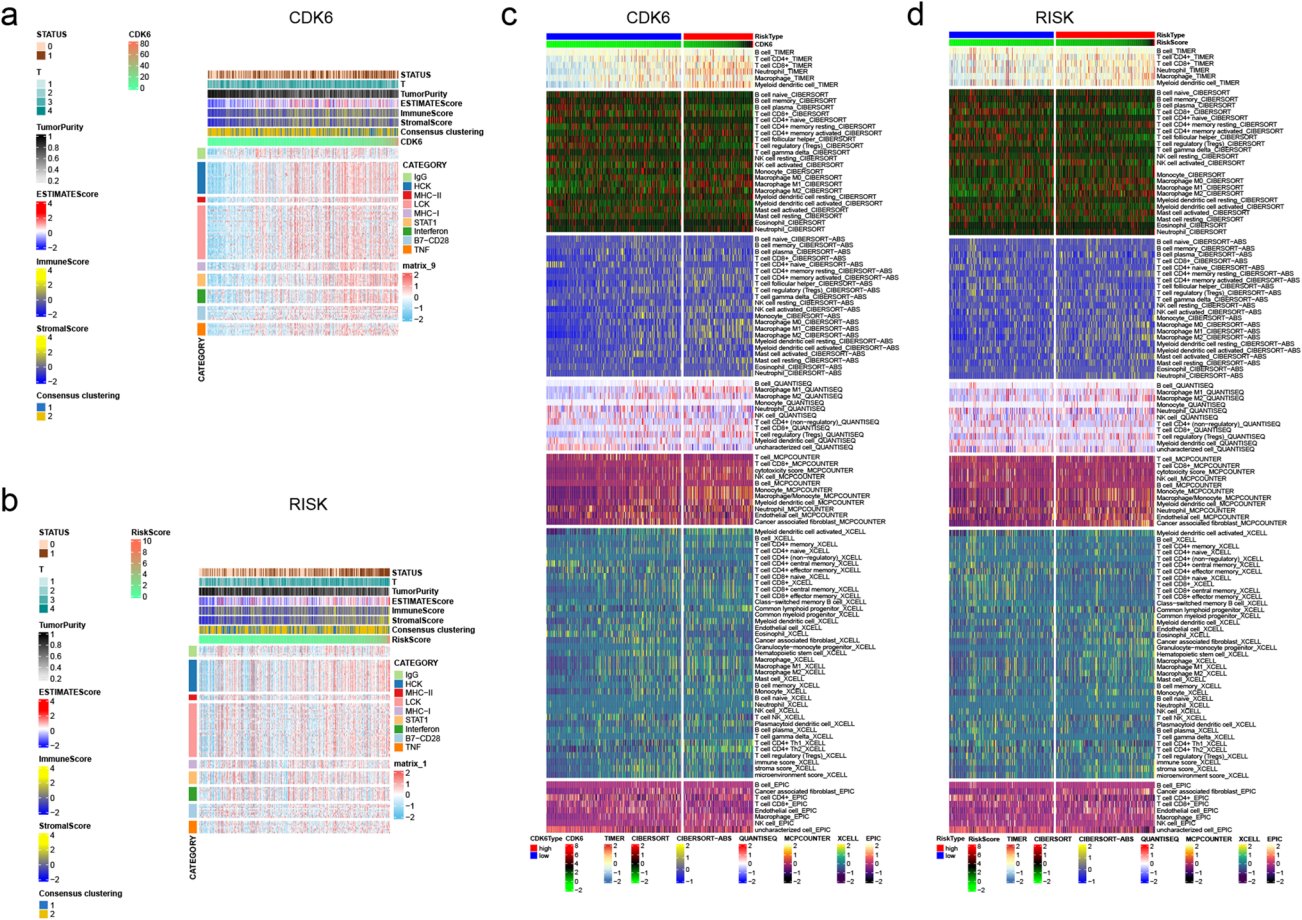
Immune microenvironment analysis. **a**, **b** Correlation of CDK6 expression or risk with status, stage, and various inflammatory factors. **c**, **d** Correlation of CDK6 expression or risk with the number of various immune cells

The heat map showed that increased CDK6 expression and risk score were associated with survival status, higher grade, estimate, stromal and immune scores, and inflammatory factor expression (Fig. 8a, b). The R package “pheatmap” was used to determine the relationship between immune cells and CDK6 expression and risk score. The number of M2 macrophages increased with higher CDK6 expression and risk score (Fig. 8c, d).

### Identification of sensitive drugs

**Fig. 9:**
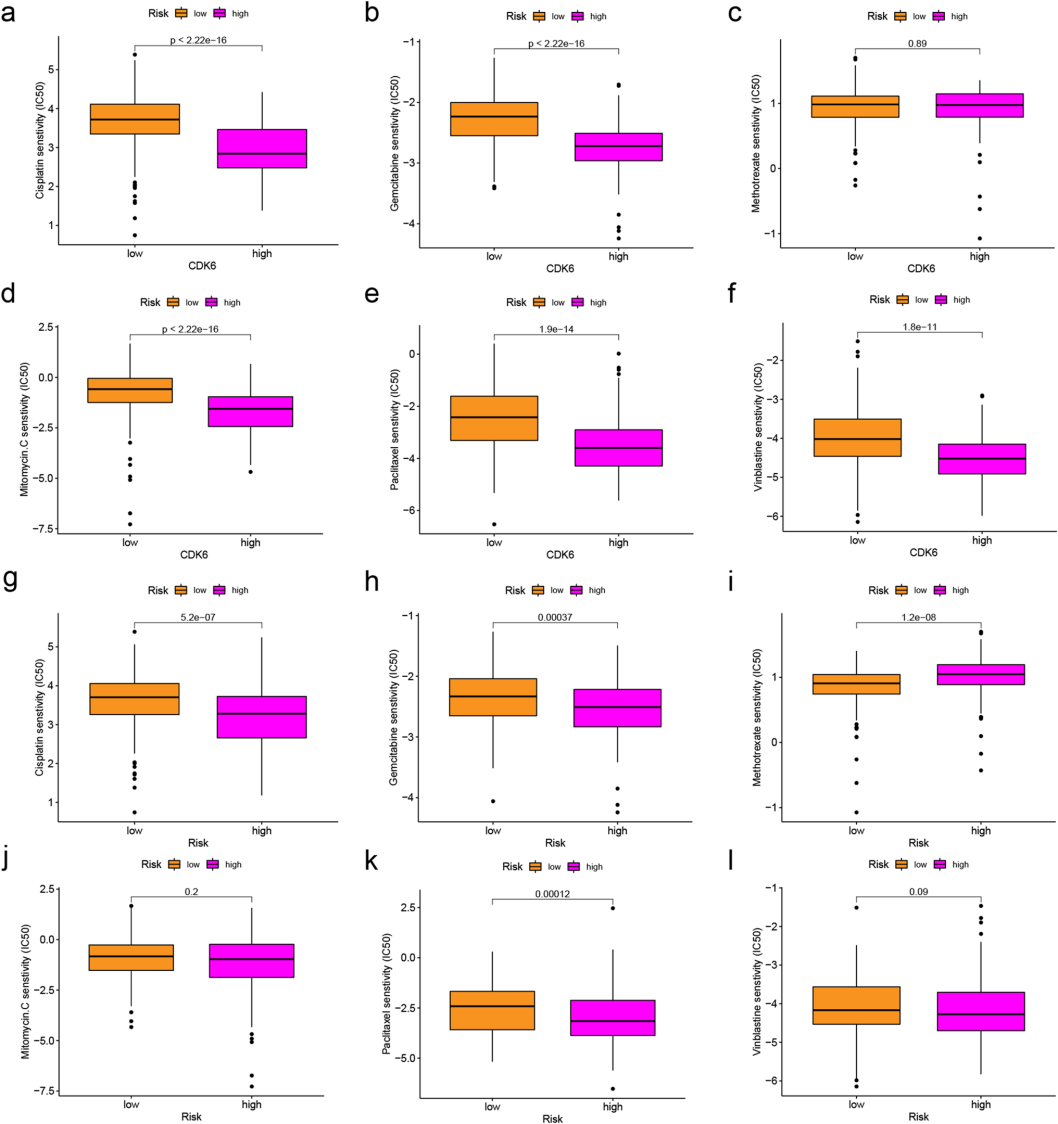
Drug sensitivity analysis. **a**–**f** Different CDK6 expression patients’ sensitivity to cisplatin (*P* < 0.001), gemcitabine (*P* < 0.001), methotrexate (*P* > 0.05), mitomycin C (*P* < 0.001), paclitaxel (*P* < 0.001), and vinblastine (*P* < 0.001). **g**–**l** Different risk patients’ sensitivity to cisplatin (*P* < 0.001), gemcitabine (*P* < 0.001), methotrexate (*P* > 0.05), mitomycin C (*P* > 0.05), paclitaxel (*P* < 0.01), and vinblastine (*P* > 0.05)

After converting gene expression into drug sensitivity data using the R package, patients were divided into high- or low-expression levels or high- or low-risk according to CDK6 and risk score. Then sensitive therapeutic agents were sought. The high-CDK6 expression group’s sensitivities to cisplatin (Fig. 9a), gemcitabine (Fig. 9b), mitomycin C (Fig. 9d), paclitaxel (Fig. 9e), and vinblastine (Fig. 9f) were higher than those of the low-expression group (*P* < 0.001 for all). There is no different sensitivity to methotrexate between the high- and low-CDK6 expression group (Fig. 9c). The high-risk group’s sensitivities to cisplatin (Fig. 9g), gemcitabine (Fig. 9h), and paclitaxel (Fig. 9k) were higher than those of the low-risk group (*P* < 0.001 for all). For methotrexate (Fig. 9i), the low-risk group was more sensitive (*P* < 0.001). The high- and low-risk groups have no different sensitivity to mitomycin C (Fig. 9j) and vinblastine (Fig. 9l).

### CDK6 functional experiments

**Fig. 10:**
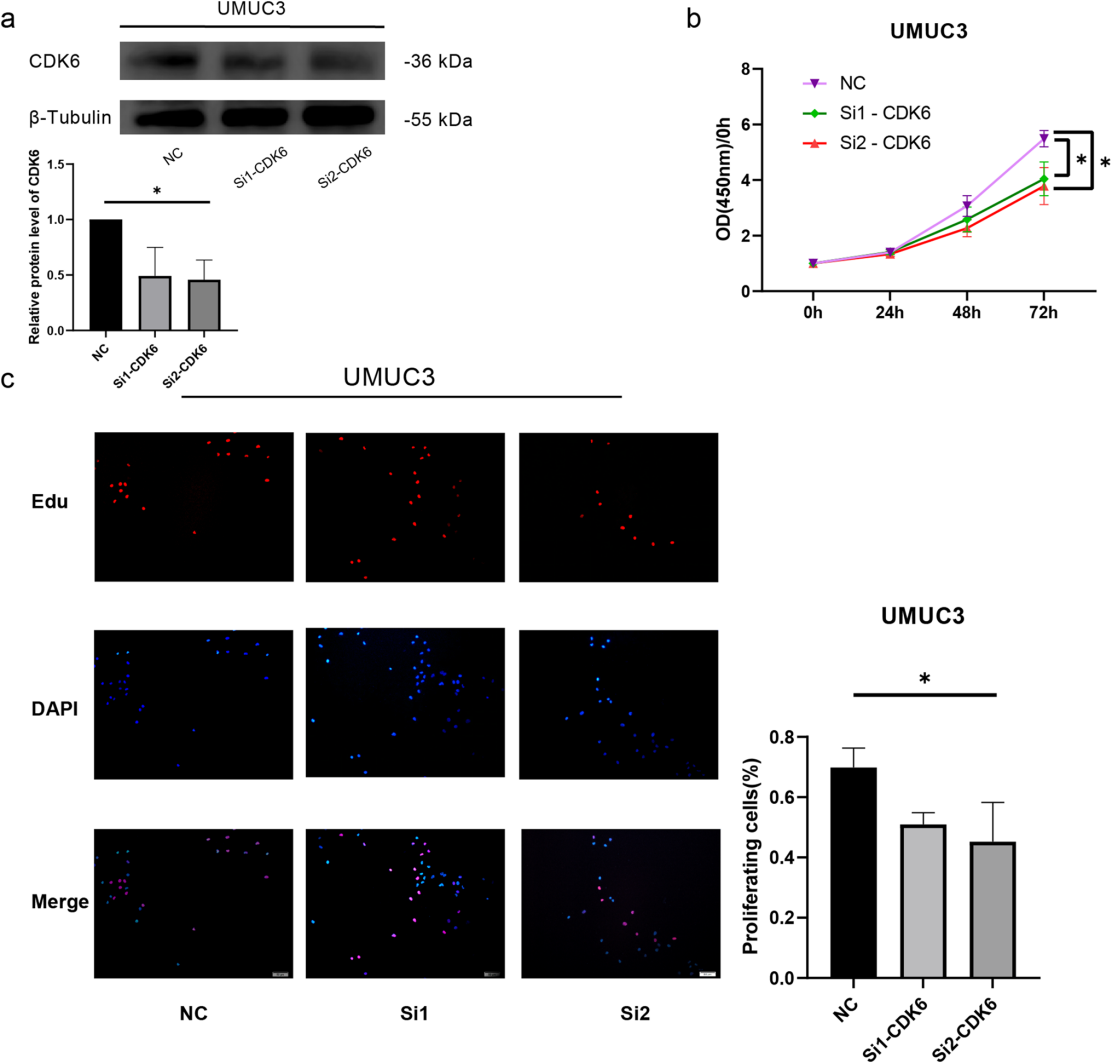
Functional analysis of CDK6 in BLCA cells. **a** Relative protein level of CDK6 in UMUC3 cells after CDK6 was knockdown. β-Tubulin served as loading control. **b** The effect of CDK6 on the proliferation of UM-UC-3 cells was measured using a CCK-8 assay. **c** The effect of CDK6 on the proliferation of UM-UC-3 cells was observed using an EdU assay. To determine whether there was a significant difference between groups, the *t* test was used and was expressed as the mean ± standard deviation

For validation of the bioinformatics results in vitro, we selected the risk factor CDK6. Changes in BLCA cell proliferation were observed by decreasing CDK6 expression using siRNA (Fig. 10a). In the CCK8 assay, the proliferation of cells from the CDK6 knockdown group was lower than the normal group (Fig. 10b). The results in the EdU experiment were consistent with those of the CCK8 experiment (Fig. 10c).

## Discussion

Bladder urothelial cancer is a heterogeneous malignancy with a high likelihood of incidence and recurrence (Robertson et al. 2017; Lindskrog et al. 2021). Despite developing comprehensive treatment strategies for BLCA, there is still a lack of markers that can effectively diagnose it and guidance for molecular targeted therapy and individualized treatment. Based on PI3K pathway-related genes in this study, we divided BLCA patients into two subtypes using the NMF algorithm. Twenty-five co-expression modules were then identified with WGCNA analysis, with the brown and green modules having the highest correlation with clusters 1 and 2. The biological functions and pathways that could be affected by the two modules were then analyzed.

We developed a prognostic score model from transcriptome data using TCGA-BLCA and validated its stability in an external cohort using univariate Cox, LASSO-Co, and multivariate Cox analyses. This model included seven prognostic factors (CDK6, EGFR, IGF1, ITGB7, PDGFRA, RPS6, and VWF). The patients were divided into subgroups according to their stage, age, and sex to test whether the model would determine the outcome. The nomogram based on clinical parameters and risk had better predictive ability than risk and independent clinical parameters. We found that M2 macrophages highly correlated with CDK6 and risk, and CDK6 and risk were associated with status, T-stage, estimate score, immune score, stromal score, and various inflammatory factors. A prognostic model of five lncRNAs was established using univariate and multivariate Cox proportional hazard regression and has an excellent predictive ability (Liu et al. 2022). After screening prognostic factors using univariate regression analysis, an eight-m5C-related LncRNA risk model was constructed using the LASSO-Cox regression method to predict outcomes (Yuan et al. 2021). Univariate and LASSO-Cox regression analyses were used to establish an 11-lncRNA signature for predicting outcomes, and then, the signature was validated in all tests (Gao et al. 2021). A novel immune-related lncRNA signature was also constructed using the univariate and LASSO-Cox regression method. The AUCs of the signature were high, demonstrating the excellent ability to predict outcomes (Liu et al. 2021). The method of our constructed model was similar to the methods mentioned above. Our model was established using multivariate Cox regression analysis after screening genes associated with outcomes using univariate and LASSO-Cox regression analysis. A series of evaluations of the model showed that it also has an excellent ability to predict patients’ prognoses.

CDK6 overexpression has been found in several cancers, and tumor drug resistance increases when CDK6 expression is elevated (Tadesse et al. 2015; Yang et al. 2017; Li et al. 2018; Cornell et al. 2019). Upregulation of ODK6 promotes the proliferation of colon cancer (Liu et al. 2020), breast cancer (Leung and Potter 1987; Sherr 1996), and human hepatocellular carcinoma (Guo et al. 2018). Lanaya et al. suggested that EGFR can promote hepatocellular carcinoma (Lanaya et al. 2014), and Feng et al. suggested that EGFR can promote renal cell carcinoma (Feng et al. 2017). ITGB7 is involved in regulating adhesion (Ohguchi et al. 2016) and proliferation and glucose metabolism in cervical cancer (Chai et al. 2019).

IGF1 affects the incidence and mortality of tumors (Levine et al. 2014) and survival from breast tumors (Zhang et al. 2009). PDGFRA may be an anti-cancer drug target in gastrointestinal cancer (Yin et al. 2020). RPS6 may be an independent prognostic marker for metastatic renal cell carcinoma (Fang et al. 2017). Aryal et al. suggested that VWF may be an independent predictor of early hepatocellular carcinoma recurrence (Aryal et al. 2019) and be negatively related to ovarian, bladder, and colon cancers (Wang et al. 2005).

Immune cell expression analysis indicated that CDK6 expression level and risk positively correlated with M2 macrophages, which promote tumor development, progression (Zhao et al. 2020), and poor outcome (e.g., in colon cancer (Cheng et al. 2018)). The inflammatory response can accelerate cancer progression (Colaprico et al. 2020). IgG mediates inflammatory responses, promoting tumor metastasis (Stamatiades et al. 2016) (e.g., in pancreatic cancer (Chen et al. 2019)). Excessive release of pro-inflammatory cytokines promotes the occurrence and metastasis of BLCA (Luo and Xu 2020). These findings suggest that higher CDK6 expression and risk are associated with higher IgG quantity. Our results agree with these findings.

Cisplatin and gemcitabine are first-line chemotherapeutic agents for BLCA and improve overall survival (Moufarij et al. 2003; Boulikas and Vougiouka 2004; Crabb and Douglas 2018; Hayashi et al. 2020). Methotrexate, vinblastine, and cisplatin are safe and effective adjuvant therapeutic agents for muscle-invasive BLCA (Plimack et al. 2014). Mitomycin C treats superficial BLCA and prevents recurrence and progression (Ayyildiz et al. 2007). Paclitaxel is used to treat patients with metastatic BLCA (Jacobs et al. 2010). Cisplatin, gemcitabine, mitomycin C, paclitaxel, and vinblastine may be more effective for patients with high-CDK6 expression. Cisplatin, gemcitabine, and paclitaxel may be more effective for high-risk BLCA, while methotrexate may be effective for low-risk BLCA.

Although this study relied on transcriptome data from many samples and experimental validation, it has limitations. Previous researchers provided the data, and comprehensive, in-depth clinical studies are needed before clinical application. In addition, more cohorts are needed to validate the model’s stability.

## Conclusions

We constructed a robust seven-gene prognostic risk model and validated it using an external dataset. The biological impact of genes on BLCA was identified. The number of M2 macrophages and IgG levels positively correlated with the expression of CDK6 and risk. BLCA patients were grouped based on the expression of CDK6 and risk, and drugs that may be more sensitive in different groups were identified. The proliferation ability of BLCA cells was reduced when the expression of CDK6 was reduced. CDK6 is a potential biomarker that is involved in the proliferation of BLCA. To predict outcomes in BLCA, we recommend this seven-gene signature.

## Availability of supporting data

The TCGA-BLCA dataset used in this study could be obtained from TCGA database (https://cancergenome.nih.gov/). This study’s GEO dataset (GSE32894) were obtained from the GEO database (https://www.ncbi.nlm.nih.gov/geo/).

## Supplementary Information

Below is the link to the electronic supplementary material.

Supplementary file1 (XLSX 12 KB)

Supplementary file2 (DOCX 3493 KB)

## References

[CR1] Ainsworth C (2017). Microbiome: a bag of surprises. Nature.

[CR2] Aryal B, Yamakuchi M, Shimizu T, Kadono J, Furoi A, Gejima K (2019). Bivalent property of intra-platelet VWF in liver regeneration and HCC recurrence: a prospective multicenter study. Cancer Biomark.

[CR3] Ashburner M, Ball CA, Blake JA (2000). Gene ontology: tool for the unification of biology. The Gene Ontology Consortium. Nat Genet.

[CR4] Ayyildiz A, Akgül T, Nuhoğlu B (2007) Re: Hamid Mazdak, Iraj Meshki and Fatemeh Ghassami. Effect of mitomycin C on anterior urethral stricture recurrence after internal urethrotomy. Eur Urol 51:1089–92. Eur Urol. 2007. 52(3):929 10.1016/j.eururo.2007.04.097 17499429

[CR5] Banh RS, Biancur DE, Yamamoto K (2020). Neurons release serine to support mRNA translation in pancreatic cancer. Cell.

[CR6] Berdik C (2017). Unlocking bladder cancer. Nature.

[CR7] Boulikas T, Vougiouka M (2004). Recent clinical trials using cisplatin, carboplatin and their combination chemotherapy drugs (review). Oncol Rep.

[CR8] Chai Z, Yang Y, Gu Z (2019). Recombinant viral capsid protein L2 (rVL2) of HPV 16 suppresses cell proliferation and glucose metabolism via ITGB7/C/EBPβ signaling pathway in cervical cancer cell lines. Onco Targets Ther.

[CR9] Chen Q, Wang J, Zhang Q (2019). Tumour cell-derived debris and IgG synergistically promote metastasis of pancreatic cancer by inducing inflammation via tumour-associated macrophages. Br J Cancer.

[CR10] Cheng Y, Zhu Y, Xu J (2018). PKN2 in colon cancer cells inhibits M2 phenotype polarization of tumor-associated macrophages via regulating DUSP6-Erk1/2 pathway. Mol Cancer.

[CR11] Colaprico A, Olsen C, Bailey MH (2020). Interpreting pathways to discover cancer driver genes with Moonlight. Nat Commun.

[CR12] Cornell L, Wander SA, Visal T, Wagle N, Shapiro GI (2019). MicroRNA-mediated suppression of the TGF-β pathway confers transmissible and reversible CDK4/6 inhibitor resistance. Cell Rep.

[CR13] Crabb SJ, Douglas J (2018). The latest treatment options for bladder cancer. Br Med Bull.

[CR14] Fang Y, Bao W, Rao Q, Wang X, Xia Q, Shen Q (2017). TFE3 regulates renal adenocarcinoma cell proliferation via activation of the mTOR pathway. Mol Med Rep.

[CR15] Feng ZH, Fang Y, Zhao LY, Lu J, Wang YQ, Chen ZH (2017). RIN1 promotes renal cell carcinoma malignancy by activating EGFR signaling through Rab25. Cancer Sci.

[CR16] Gao Y, Liu J, Cai B, Chen Q, Wang G, Lu Z, Jiang K, Miao Y (2021). Development of epithelial-mesenchymal transition-related lncRNA signature for predicting survival and immune microenvironment in pancreatic cancerwithexperiment validation. Bioengineered.

[CR17] Garcia-Martinez L, Zhang Y, Nakata Y, Chan HL, Morey L (2021). Epigenetic mechanisms in breast cancer therapy and resistance. Nat Commun.

[CR18] Guo J, Fang W, Sun L (2018). Ultraconserved element uc.372 drives hepatic lipid accumulation by suppressing miR-195/miR4668 maturation. Nat Commun..

[CR19] Hamm M, Sohier P, Petit V (2021). BRN2 is a non-canonical melanoma tumor-suppressor. Nat Commun.

[CR20] Hayashi K, Nikolos F, Lee YC (2020). Tipping the immunostimulatory and inhibitory DAMP balance to harness immunogenic cell death. Nat Commun.

[CR21] Hsieh DS, Wang H, Tan SW (2011). The treatment of bladder cancer in a mouse model by epigallocatechin-3-gallate-gold nanoparticles. Biomaterials.

[CR22] Jacobs BL, Lee CT, Montie JE (2010). Bladder cancer in 2010: how far have we come. CA Cancer J Clin.

[CR23] Kanehisa M, Goto S (2000). KEGG: kyoto encyclopedia of genes and genomes. Nucleic Acids Res.

[CR24] Kanehisa M, Sato Y (2020). KEGG Mapper for inferring cellular functions from protein sequences. Protein Sci.

[CR25] Lanaya H, Natarajan A, Komposch K, Li L, Amberg N, Chen L (2014). EGFR has a tumour-promoting role in liver macrophages during hepatocellular carcinoma formation. Nat Cell Biol.

[CR26] Langfelder P, Horvath S (2008). WGCNA: an R package for weighted correlation network analysis. BMC Bioinformatics.

[CR27] Leung BS, Potter AH (1987). Mode of estrogen action on cell proliferative kinetics in CAMA-1 cells. I. Effect of serum and estrogen. Cancer Invest..

[CR28] Levine ME, Suarez JA, Brandhorst S (2014). Low protein intake is associated with a major reduction in IGF-1, cancer, and overall mortality in the 65 and younger but not older population. Cell Metab.

[CR29] Li Z, Razavi P, Li Q (2018). Loss of the FAT1 tumor suppressor promotes resistance to CDK4/6 inhibitors via the Hippo pathway. Cancer Cell.

[CR30] Lindskrog SV, Prip F, Lamy P (2021). An integrated multi-omics analysis identifies prognostic molecular subtypes of non-muscle-invasive bladder cancer. Nat Commun.

[CR31] Liu S, Harmston N, Glaser TL (2020). Wnt-regulated lncRNA discovery enhanced by in vivo identification and CRISPRi functional validation. Genome Med.

[CR32] Liu J, Mei J, Wang Y, Chen X, Pan J, Tong L, Zhang Y (2021). Development of a novel immune-related lncRNA signature as a prognostic classifier for endometrial carcinoma. Int J Biol Sci.

[CR33] Liu J, Cui G, Ye J, Wang Y, Wang C, Bai J (2022). Comprehensive analysis of the prognostic signature of mutation-derived genome instability-related lncRNAs for patients with endometrial cancer. Front Cell Dev Biol.

[CR34] Luo J, Xu X (2020). Dietary fiber intake and the risk of bladder cancer in the Prostate, Lung, Colorectal and Ovarian (PLCO) cohort. Carcinogenesis.

[CR35] Moufarij MA, Phillips DR, Cullinane C (2003). Gemcitabine potentiates cisplatin cytotoxicity and inhibits repair of cisplatin-DNA damage in ovarian cancer cell lines. Mol Pharmacol.

[CR36] Ohguchi H, Hideshima T, Bhasin MK (2016). The KDM3A-KLF2-IRF4 axis maintains myeloma cell survival. Nat Commun.

[CR37] Plimack ER, Hoffman-Censits JH, Viterbo R (2014). Accelerated methotrexate, vinblastine, doxorubicin, and cisplatin is safe, effective, and efficient neoadjuvant treatment for muscle-invasive bladder cancer: results of a multicenter phase II study with molecular correlates of response and toxicity. J Clin Oncol.

[CR38] Pollak M (2018). Diet boosts the effectiveness of a cancer drug. Nature.

[CR39] Robertson AG, Kim J, Al-Ahmadie H (2017). Comprehensive molecular characterization of muscle-invasive bladder cancer. Cell.

[CR40] Sherr CJ (1996). Cancer cell cycles. Science.

[CR41] Shinde-Jadhav S, Mansure JJ, Rayes RF (2021). Role of neutrophil extracellular traps in radiation resistance of invasive bladder cancer. Nat Commun.

[CR42] Sjödahl G, Lauss M, Lövgren K (2012). A molecular taxonomy for urothelial carcinoma. Clin Cancer Res.

[CR43] Stamatiades EG, Tremblay ME, Bohm M (2016). Immune monitoring of trans-endothelial transport by kidney-resident macrophages. Cell.

[CR44] Tadesse S, Yu M, Kumarasiri M, Le BT, Wang S (2015). Targeting CDK6 in cancer: state of the art and new insights. Cell Cycle.

[CR45] Tibshirani R (1997). The lasso method for variable selection in the Cox model. Stat Med.

[CR46] Wang WS, Lin JK, Lin TC, Chiou TJ, Liu JH, Yen CC (2005). Plasma von Willebrand factor level as a prognostic indicator of patients with metastatic colorectal carcinoma. World J Gastroenterol.

[CR47] Yang C, Li Z, Bhatt T (2017). Acquired CDK6 amplification promotes breast cancer resistance to CDK4/6 inhibitors and loss of ER signaling and dependence. Oncogene.

[CR48] Yin Z, Wang Q, Yan X, Zhang L, Tang K, Cao Z (2020). Reveal the regulation patterns of prognosis-related miRNAs and lncRNAs across solid tumors in the Cancer Genome Atlas. Front Cell Dev Biol.

[CR49] Yoshihara K, Shahmoradgoli M, Martínez E (2013). Inferring tumour purity and stromal and immune cell admixture from expression data. Nat Commun.

[CR50] Yuan H, Liu J, Zhao L, Wu P, Chen G, Chen Q, Shen P, Yang T, Fan S, Xiao B, Jiang K (2021). Prognostic risk model and tumor immune environment modulation of m5C-related lncRNAs in pancreatic ductal adenocarcinoma. Front Immunol.

[CR51] Zhang Z, Kattan MW (2017). Drawing Nomograms with R: applications to categorical outcome and survival data. Ann Transl Med.

[CR52] Zhang XH, Wang Q, Gerald W (2009). Latent bone metastasis in breast cancer tied to Src-dependent survival signals. Cancer Cell.

[CR53] Zhao Z, Ukidve A, Kim J, Mitragotri S (2020). Targeting strategies for tissue-specific drug delivery. Cell.

