# Supplementary material for: In silico development and experimental validation of a novel 7-gene signature based on PI3K pathway-related genes in bladder cancer
Source: Funct Integr Genomics. 2022 Jul 28;22(5):797–811. doi: 10.1007/s10142-022-00884-2 (PMC9550739; doi:10.1007/s10142-022-00884-2)
Supplement: Supplementary file 2 — Supplementary file2 (DOCX 3493 KB) [file 10142_2022_884_MOESM2_ESM.docx]

**Fig. S1**


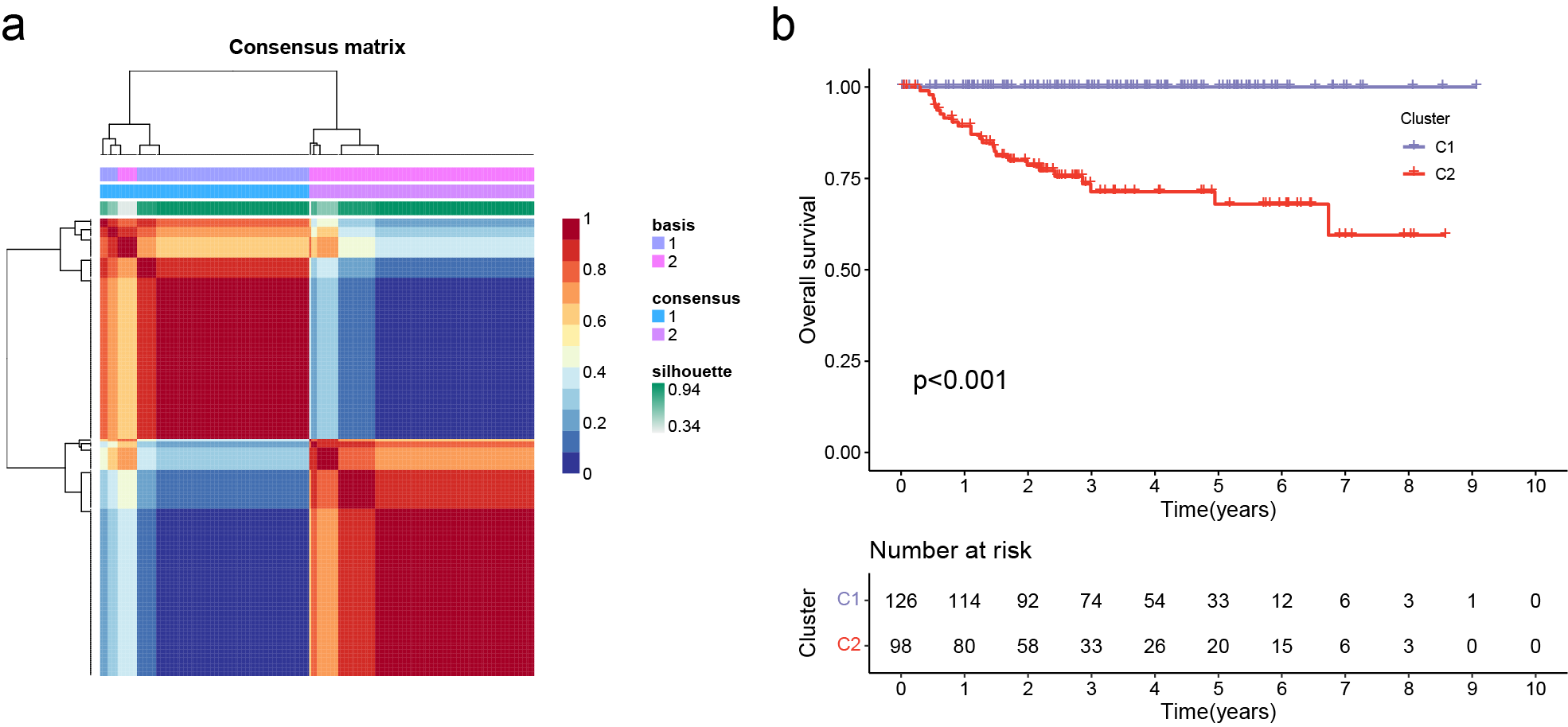


**Fig. S2**

**
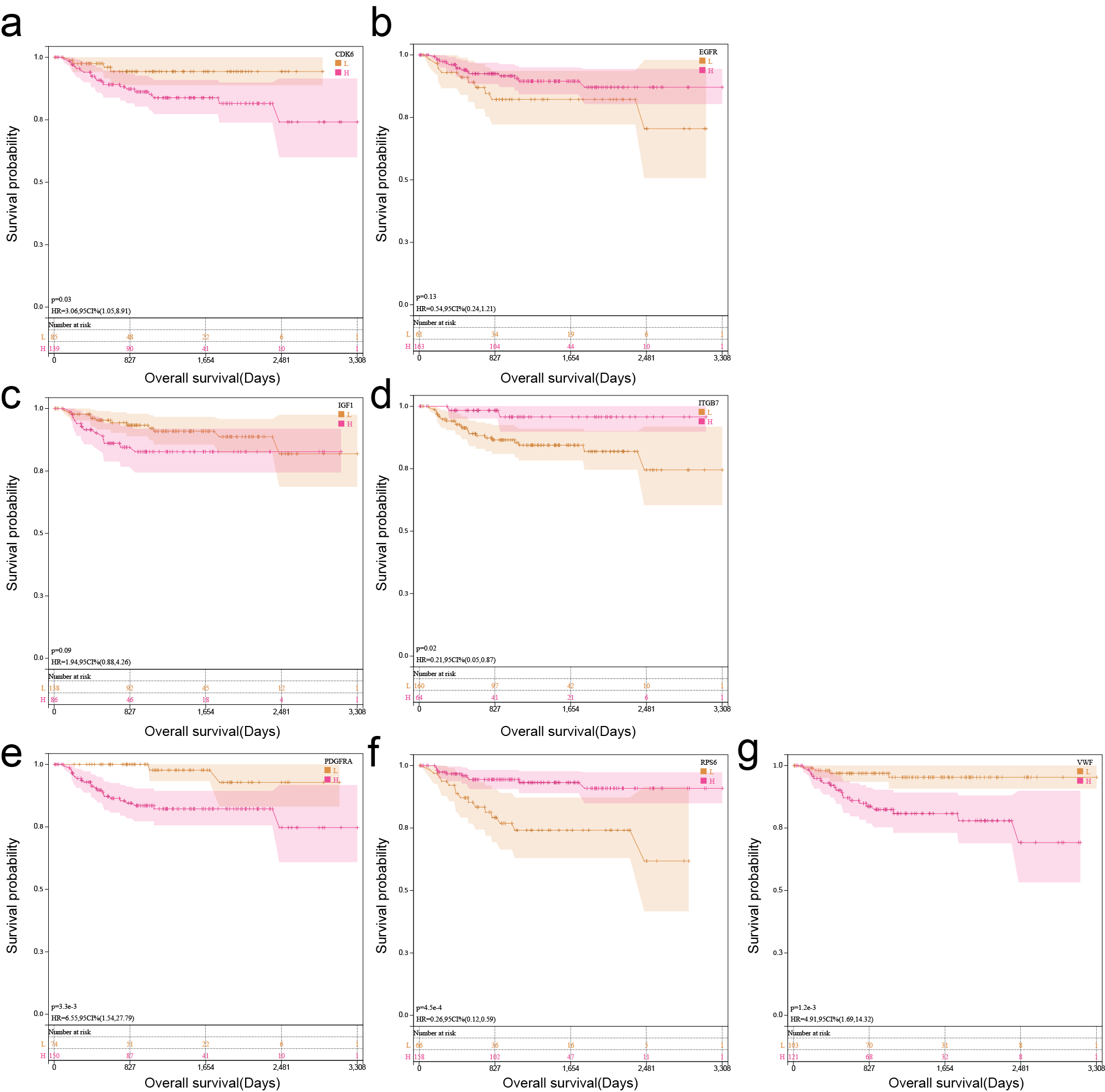
**

**Fig. S3**

**
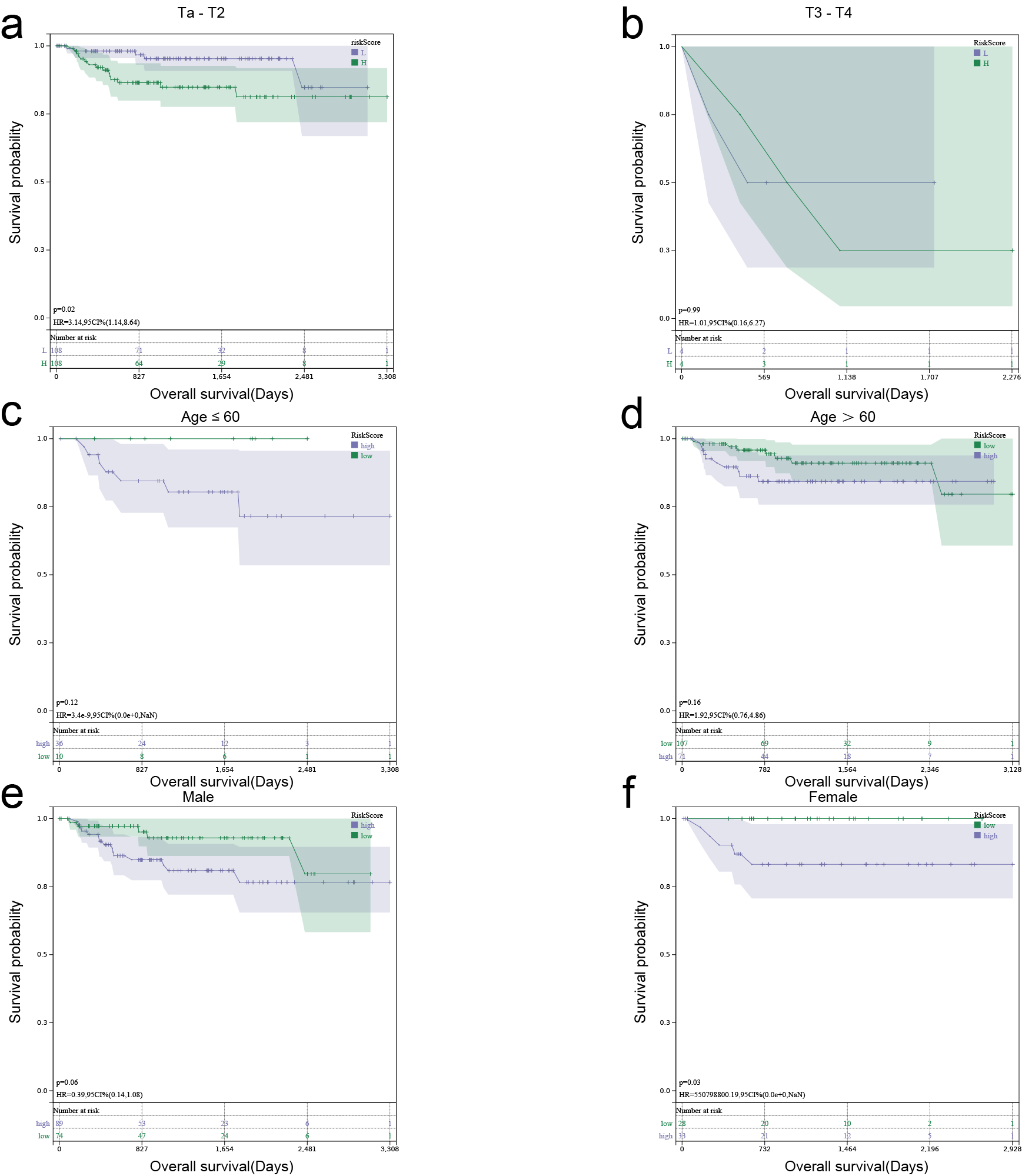
**

**Fig. S1**: Non-negative matrix factorization (NMF) analysis (a) NMF clustering (K = 2) consensus map (b) The two subtypes' overall survival.

**Fig. S2**: (a-g) Kaplan-Meier survival curves of high and low-expression groups based on the expression of seven genes in the model in GSE32894.

**Fig. S3**: Kaplan-Meier (KM) survival analysis in different subgroups based on stage, age, and gender in GSE32894 (a) KM survival curves of patients in Ta-T2 (P = 0.02). (b) KM survival curves of patients in T3-T4 (P>0.05). (c) KM survival curves of patients aged ≤ 60 (P=0.12). (d) KM survival curves of patients aged > 60 (P=0.16). (e) KM survival curves of male patients (P=0.06). (f) KM survival curves of female patients (P=0.03).
